# Energy intake and expenditure in patients with Alzheimer’s disease and mild cognitive impairment: the NUDAD project

**DOI:** 10.1186/s13195-020-00687-2

**Published:** 2020-09-26

**Authors:** Astrid S. Doorduijn, Marian A. E. de van der Schueren, Ondine van de Rest, Francisca A. de Leeuw, Heleen M. A. Hendriksen, Charlotte E. Teunissen, Philip Scheltens, Wiesje M. van der Flier, Marjolein Visser

**Affiliations:** 1 Department of Nutrition and Dietetics, Amsterdam UMC, Vrije Universiteit Amsterdam, Amsterdam Public Health research institute, PO Box 7057, 1007 MB Amsterdam, The Netherlands; 2 Alzheimer Center Amsterdam, Department of Neurology, Amsterdam Neuroscience, Vrije Universiteit Amsterdam, Amsterdam UMC, PO Box 7057, 1007 MB Amsterdam, The Netherlands; 3 Division of Human Nutrition and Health, Wageningen University & Research, Wageningen, The Netherlands; 4 Department of Nutrition and Health, HAN University of Applied Sciences, Nijmegen, The Netherlands; 5 Neurochemistry Laboratory, Department of Clinical Chemistry, Amsterdam Neuroscience, Vrije Universiteit Amsterdam, Amsterdam UMC, Amsterdam, The Netherlands; 6 Department of Health Sciences, Faculty of Science, Vrije Universiteit Amsterdam and the Amsterdam Public Health research institute, Amsterdam, The Netherlands

**Keywords:** Malnutrition, Dementia, Older adults, Dietary intake, Physical activity

## Abstract

**Background:**

Malnutrition is common in patients with Alzheimer’s disease (AD) dementia and mild cognitive impairment (MCI) and is associated with institutionalization and increased mortality. Malnutrition is the result of a negative energy balance, which could be due to reduced dietary intake and/or higher energy expenditure. To study underlying mechanisms for malnutrition, we investigated dietary intake and resting energy expenditure (REE) of patients with AD dementia, MCI, and controls. In addition, we studied associations of global cognition (Mini-Mental State Examination (MMSE)) and AD biomarkers with dietary intake and REE.

**Methods:**

We included 219 participants from the NUDAD project, 71 patients with AD dementia (age 68 ± 8 years, 58% female, MMSE 24 ± 3), 52 with MCI (67 ± 8 years, 42% female, MMSE 26 ± 2), and 96 controls (62 ± 7 years, 52% female, MMSE 28 ± 2). We used a 238-item food frequency questionnaire to assess dietary intake (energy, protein, carbohydrate, and fat). In a subgroup of 92 participants (30 patients with AD dementia, 22 with MCI, and 40 controls) we measured REE with indirect calorimetry. Between-group differences in dietary intake and REE were tested with ANOVAs. In the total sample, linear regression analyses were used to explore potential associations of MMSE score and AD biomarkers with dietary intake and REE. All analyses were adjusted for age, sex, education, and body mass index or fat-free mass.

**Results:**

Patients with AD dementia and MCI did not differ from controls in total energy intake (1991 ± 71 and 2172 ± 80 vs 2022 ± 61 kcal/day, *p* > 0.05) nor in protein, carbohydrate, or fat intake. Patients with AD dementia and MCI had a higher REE than controls (1704 ± 41 and 1754 ± 47 vs 1569 ± 34 kcal/day, *p* < 0.05). We did not find any association of MMSE score or AD biomarkers with dietary intake or REE.

**Conclusions:**

We found a higher REE, despite similar energy intake in patients with AD and MCI compared to controls. These findings suggest that elevated metabolism rather than reduced energy intake explains malnutrition in AD. These results could be useful to optimize dietary advice for patients with AD dementia and MCI.

## Background

Malnutrition in patients with Alzheimer’s disease (AD) dementia is associated with a higher rate of institutionalization and increased mortality [1, 2]. We previously found that even patients with mild AD dementia and mild cognitive impairment (MCI) have a worse nutritional status, assessed with the Mini Nutritional Assessment (MNA) [3], than controls [4]. Malnutrition reflects a negative energy balance, which can arise from a reduced energy intake, a higher energy expenditure, or a combination of both. To date, it is unclear what causes the negative energy balance in patients with MCI or AD dementia. It is conceivable that impaired memory affects dietary intake [5], which would fit with the notion of a reduced dietary intake as a result of cognitive decline. Alternatively, one could hypothesize that dietary intake or energy expenditure change as an effect of Alzheimer-related brain changes, reflected by AD biomarkers in cerebrospinal fluid (CSF).

Forgetting to eat, no longer being able to use eating utensils, or lack of appetite are hypothesized to cause a reduced dietary intake in patients with AD dementia [5, 6]. However, only one study found that energy intake of patients with AD dementia was lower than that of controls [7], while other studies found no differences in energy intake between patients with AD dementia and controls [8–10]. This inconsistency might be caused by different dietary assessment methods and different inclusion criteria for patients with AD dementia and controls. Resting energy expenditure (REE) accounts for about 70% of daily expenditure and is hypothesized to be higher in patients with AD dementia than controls [6]. This is supported by one study [11]; however, two other studies found no differences in REE between patients with AD dementia and controls [8, 12]. Furthermore, in patients with AD dementia, total energy expenditure might also be elevated due to a higher physical activity level, possibly due to wandering [13]. Contrarily, studies that measured physical activity found that patients with AD dementia and MCI were actually less physically active and performed their activities at lower intensities than controls [14, 15].

Thus, the results of studies focusing on aspects of energy balance are conflicting, probably due to the broad range of AD severity within the studies. Moreover, patients with MCI were not included in studies that assessed dietary intake, and in only one study measuring physical activity, while changes in nutritional status already occur in pre-dementia stages [4]. Therefore, the aim of this paper is to compare dietary intake, REE, and physical activity of patients with AD dementia and MCI with cognitively normal controls. In addition, we assessed associations between global cognition and AD biomarkers with dietary intake and REE.

## Methods

### Participants

The NUDAD (Nutrition, the Unrecognized Determinant in Alzheimer’s Disease) study is a prospective cohort study, investigating nutritional determinants in AD dementia and pre-dementia stages, with a 3-year clinical follow-up [4]. The total NUDAD cohort includes 552 participants of the Amsterdam Dementia Cohort who visited the Alzheimer center between September 2015 and August 2017 and were diagnosed with AD dementia, MCI, or subjective cognitive decline (SCD) and had a Mini-Mental State Examination (MMSE) score > 16 [16]. All patients underwent a standardized dementia screening, including medical history and neurological examination, neuropsychological assessment, and laboratory tests [17]. Patients were diagnosed with MCI and probable AD dementia according to the corresponding National Institute on Aging-Alzheimer’s Association criteria [18, 19]. As controls, we used individuals with SCD, who presented with memory complaints but performed normal on all clinical examinations (i.e., criteria for MCI, dementia or any other neurological or psychiatric disorder that could explain their cognitive complaints not met) [17]. For the current study, data of 225 participants who filled in a food frequency questionnaire (FFQ) [20] were used. They were representative for the total NUDAD cohort (*n* = 552) regarding age, sex, MMSE score, and body mass index (BMI).

Of the 225 participants, a subgroup of 92 participated in additional measurements of REE, physical activity, and a 3-day food diary. For this subgroup, inclusion criteria were age ≥ 50 years, MMSE score ≥ 19, being medically stable (assessed by physician), and having sufficient knowledge of the Dutch language. Exclusion criteria were being a current smoker, having a major psychiatric disorder, having neurological disorders other than AD known to influence smell and taste, having a severe food allergy or severe disease of the digestive tract, or being recently diagnosed with cancer other than basal cell carcinoma of the skin.

### Dietary intake

Two dietary assessment methods were used: the HELIUS FFQ (*n* = 225) and a 3-day food diary in the subgroup (*n* = 92). The FFQ provides general information about habitual intake, whereas the food diary provides detailed information about dietary intake on specific days. The methods therefore provide complementary information. The FFQ is a self-administrated questionnaire asking about the frequency, amount, and type of 238 items consumed in the past month [20]. On the 3-day food diary, participants report their dietary intake on three consecutive days including one weekend day and includes all meals, snacks, and beverages consumed. The diaries were cross-checked with the participant by a qualified nutritionist to ensure that they were complete and of sufficient detail. Dietary data were coded in the software programme Compl-eat (http://www.compleat.nl) [21]. For both the FFQ and the 3-day food diary, daily intakes of food items and their nutrients were calculated using the Dutch food composition table 2013 [22]. A dietician performed a data check: the highest and lowest percentiles for energy, protein, fruit, and vegetable intake were checked for errors in amount or coding. Participants were excluded if more than ten items were missing on the FFQ or if they reported an implausible energy intake of < 500 or > 3500 kcal for women and < 800 or > 4000 kcal for men on the FFQ or 3-day food diary [23]. On the FFQ, two participants had more than ten items missing and four reported an implausible energy intake, leaving 219 participants for data analysis. No participants reported an implausible energy intake on the 3-day food diary. For both the FFQ and food diary, daily energy intake in kcal as well as protein, carbohydrate, and fat intake in energy percentage (EN%) were derived.

### Energy expenditure

Daily REE of participants in the subgroup was assessed in fasting state (> 4 h) using indirect calorimetry (Quark RMR, COSMED Benelux BV, the Netherlands) for a duration of 30 min. Data from the first 5 min were automatically removed. Participants were placed in supine position with the ventilated hood over their head. Oxygen consumption and carbon dioxide production were analyzed continuously and converted into energy expenditure with the Weir equation [24]. One participant suffered from hyperventilation during the measurement, and three participants were not in a fasted state, leaving data of 88 participants for analysis.

In a subgroup, time spent physically active was measured using ActiGraph accelerometers (model wGT3X-BT, ActiGraph Inc., Pensacola, USA). The accelerometer was attached to a tight elastic belt and worn around the waist on the right side for 1 week. Participants were instructed to wear the accelerometer during waking hours and during all activities that do not involve water (e.g., showering). Data available of at least four valid days (> 10 h wear time per day) was downloaded and processed using the manufacturer’s software (Actilife v6.13.4, ActiGraph Pensacola, USA) [25]. Forty-four participants returned the accelerometer, two of them did not wear it due to sickness, six had less than four valid days, and five had less than 10 h wear time per day, leaving data of 31 participants for analysis. Based on the number of counts per minute (c/m), physical activity was measured in separate intensity categories: light (100–2019 c/m), which was further subdivided into low-light (100–759 c/m, e.g., light household activities) and high-light (760–2019 c/m, e.g., walking), and moderate to vigorous (> 2020 c/m, e.g., cycling) [25]. Total physical activity was the sum of light and moderate to vigorous physical activity.

### Determinants

Global cognition was measured with the MMSE, with a maximum score of 30 and a higher score indicating better global cognition [16]. AD biomarkers β-amyloid 42 (Aβ_42_), total tau (tau), and phosphorylated tau (p-tau) were measured in cerebrospinal fluid (CSF). CSF was obtained by lumbar puncture using a 25-gauge needle and collected in 10 ml polypropylene tubes (Sarstedt) following standardized protocols [26]. Aβ_42_, tau, and p-tau concentrations were determined with sandwich ELISAs (Fujirebio, Ghent, Belgium) [27]. Aβ_42_ concentrations were adjusted for the drift that occurred over the years [28], and data were available of 148 participants.

### Other variables

Descriptive characteristics included the following: age, sex, BMI from measured weight and height (kg/m^2^), level of education, and living situation (with partner/children, alone). Level of education was assessed using the Verhage classification system [29] and categorized into low (scores 1–3), intermediate (scores 4 and 5), and high (scores 6 and 7). Fat-free mass (FFM, kg) was estimated using bio-electrical impedance analysis (Bodystat Quadscan 4000) and the formula of Kyle [30] and available of 195 participants. Furthermore, nutritional status was evaluated with the Mini Nutritional Assessment (MNA) [3]. To avoid that differences in MNA score were driven by differences in cognitive performance, we excluded the item on neuropsychological problems. MNA score range from 0 to 28 with a higher score indicating a better nutritional status and available of 163 participants.

### Statistical analyses

We compared participant characteristics, dietary intake, and energy expenditure between diagnosis groups using ANOVA with post-hoc Bonferroni adjusted *t* tests and chi-square tests where appropriate. ANOVAs of dietary intake were adjusted for age, sex, education, and BMI; for REE, ANOVAs were adjusted for FFM instead of BMI, and ANOVAs of physical activity were adjusted for age, sex, and education. We performed sensitivity analyses restricted to participants with a confirmed CSF biomarker profile for the dietary intake and REE analyses. In the total sample, linear regression analyses were used to explore potential associations of MMSE score and each AD biomarker (independent variables) with dietary intake or REE (dependent variables in separate models). The associations of MMSE and AD biomarkers with dietary intake were adjusted for age, sex, education, and BMI, whereas the associations with REE were adjusted for age, sex, education, and FFM. Significance was set at *p* value < 0.05. All statistical analyses were performed with Statistical Package for the Social Sciences (SPSS Inc., Chicago, IL) version 24.0 for Windows.

## Results

Table 1 presents characteristics of the 219 participants with complete dietary intake data assessed by the FFQ (supplementary table A shows study sample characteristics for 3-day food diary, REE and physical activity). Patients with AD dementia and MCI were older and had a lower MMSE score compared to controls, and patients with AD dementia were lower educated than patients with MCI and controls. Patients with AD dementia had lower Aβ_42_ levels and higher tau and p-tau levels than controls. Twenty-one controls and five patients with AD dementia did not have a matching CSF biomarker profile. Groups did not differ in BMI or MNA score.

**Table 1:** Characteristics of total study sample with FFQ available (*n* = 219) according to diagnosis group

	Controls		MCI		AD dementia	
	*N*		*N*		*N*	
Age (years)	96	61.8 ± 7.0	52	66.9 ± 8.1^a^	71	67.9 ± 8.2^a^
Sex, female	96	50 (52.1)	52	22 (42.3)	71	41 (57.7)
MMSE score	96	28.4 ± 1.5	52	26.4 ± 2.3^a^	71	23.5 ± 3.0^a,b^
BMI (kg/m^2^)	96	25.9 ± 4.5	52	25.6 ± 3.6	71	25.1 ± 4.3
FFM (kg)	89	53.0 ± 11.5	44	52.9 ± 9.1	62	50.3 ± 10.2
MNA score	70	24.7 ± 2.2	41	24.5 ± 2.8	52	24.1 ± 2.2
Level of education	96		52		71	
Low		1 (1.0)		4 (7.7)^a^		4 (5.6)^a^
Intermediate		36 (37.5)		23 (44.2)^a^		36 (50.7)^a^
High		59 (61.5)		25 (48.1)^a^		31 (43.7)^a^
Living situation	96		52		71	
With partner/children		76 (79.2)		45 (86.5)		56 (78.9)
Alone		20 (20.8)		7 (13.5)		15 (21.1)
**AD biomarkers**						
Aβ_42_ (pg/ml)	62	963 ± 302	37	849 ± 325	49	595 ± 170^a,b^
Tau (pg/ml)	61	355 ± 315	37	518 ± 293	49	784 ± 376^a,b^
P-tau (pg/ml)	61	53 ± 32	37	71 ± 30^a^	49	93 ± 35^a,b^

Data in mean ± SD; *n* (%)

*AD* Alzheimer’s disease, *MCI* mild cognitive impairment, *MMSE* Mini-Mental State Examination, *FFQ* food frequency questionnaire, *BMI* body mass index, *FFM* fat free mass, *MNA* Mini Nutritional Assessment without item on neuropsychological problems, *Aβ*_*42*_ β-amyloid 42, *p-tau* phosphorylated tau

^a^Significantly different from controls upon post-hoc testing

^b^Significantly different from MCI upon post-hoc testing

### Dietary intake

Energy intake assessed by FFQ was 1991 ± 71 kcal/day for patients with AD dementia, 2172 ± 82 kcal/day for patients with MCI, and 2022 ± 61 kcal/day for controls and did not differ across groups (Table 2). When we analyzed specific macronutrients, groups also did not differ in protein, carbohydrate, or fat intakes. Similarly, energy, protein, carbohydrate, and fat intake assessed with the 3 day-food diary did not differ across groups. Restricting the analysis to participants with a confirmed biomarker profile showed similar results (Supplementary table B). Adjusted linear regression analyses showed no associations of MMSE score or AD biomarkers with dietary intake by FFQ (Table 3).

**Table 2:** Dietary intake and energy expenditure according to diagnosis group

	Controls	MCI	AD dementia	***p*** value
**Dietary intake**				
*FFQ*, *N*	96	52	71	
Energy (kcal/day)	2022 ± 61	2172 ± 80	1991 ± 71	0.196
Protein (EN%)	15.2 ± 0.3	15.5 ± 0.4	15.1 ± 0.3	0.688
Carbohydrate (EN%)	41.4 ± 0.7	41.2 ± 1.0	40.2 ± 0.8	0.572
Fat (EN%)	34.5 ± 0.6	34.5 ± 0.8	34.2 ± 0.7	0.948
*3-day food diary*, *N*	40	22	30	
Energy (kcal/day)	2081 ± 71	2042 ± 93	2000 ± 83	0.787
Protein (EN%)	17.0 ± 0.6	15.8 ± 0.7	15.2 ± 0.6	0.079
Carbohydrate (EN%)	40.5 ± 1.2	42.6 ± 1.5	42.0 ± 1.4	0.540
Fat (EN%)	35.0 ± 1.0	36.0 ± 1.3	37.1 ± 1.2	0.424
**Energy expenditure**				
*Resting energy expenditure*, *N*	38	22	28	
*Fasted (hours)*	14.1 ± 0.3	14.6 ± 0.4	13.7 ± 0.4	0.308
Oxygen consumption (ml/min)	229 ± 5	257 ± 7^a^	248 ± 6	**0.007**
Carbon dioxide production (ml/min)	184 ± 4	201 ± 6	188 ± 5	0.076
REE (kcal/day)	1578 ± 34	1762 ± 48^a^	1691 ± 43^a^	**0.009**
REE (kcal/kg FFM)	30.8 ± 0.7	34.5 ± 0.9^a^	33.2 ± 0.8	**0.007**
*Physical activity*, *N*	11	10	10	
Wear time (min/day)	881.9 ± 23.1	868.5 ± 26.4	869.7 ± 24.5	0.911
Total (min)	328.5 ± 28.3	358.5 ± 32.3	302.8 ± 30.0	0.505
Light (min)	305.3 ± 26.4	322.9 ± 30.1	272.0 ± 28.0	0.486
Low light (min)	223.8 ± 16.5	222.5 ± 19.8	201.5 ± 17.6	0.613
High light (min)	81.5 ± 15.4	100.4 ± 17.6	70.4 ± 16.3	0.514
Moderate to vigorous (min)	15.0 [2.9–45.1]	22.7 [4.5–51.0]	25.3 [12.6–54.2]	0.461

Data in mean ± SE; median [interquartile range]; intake: ANOVA adjusted for age, sex, education, and BMI; REE: ANOVA post-hoc Bonferroni adjusted for age, sex, education, and FFM; physical activity: ANOVA adjusted for age, sex, and education

*AD* Alzheimer’s disease, *MCI* mild cognitive impairment, *FFQ* food frequency questionnaire, *EN%* energy percentage, *FFM* fat free mass, *REE* resting energy expenditure

^a^Significantly different from controls upon post-hoc testing

**Table 3:** Associations of global cognition and AD biomarkers with dietary intake and REE

		Dietary intake					Energy expenditure	
		***N***	Energy (kcal/day)	Protein (EN%)	Carbohydrate (EN%)	Fat (EN%)	***N***	REE (kcal/day)
Global cognition	MMSE	219	− 2.63 (− 29.10;23.85)	0.23 (− 0.08; 0.54)	− 0.08 (− 0.34; 0.71)	− 0.32 (− 1.24; 0.60)	88	− 11.05 (− 27.00; 4.90)
AD biomarkers	Aβ_42_	148	0.10 (− 0.19; 0.39)	0.00 (− 0.00; 0.00)	0.00 (− 0.00; 0.01)	0.00 (− 0.00; 0.00)	54	− 0.05 (− 0.27; 0.18)
	Tau	148	0.08 (− 0.17; 0.32)	0.00 (− 0.00; 0.00)	− 0.00 (− 0.00; 0.00)	0.00 (− 0.00; 0.00)	54	0.02 (− 0.15; 0.19)
	P-tau	148	0.51 (− 1.99; 3.01)	0.01 (− 0.01; 0.02)	− 0.01 (− 0.04; 0.03)	0.00 (− 0.03; 0.03)	54	0.27 (− 1.50; 2.03)

Data presented as *β* (95% CI) (regression coefficient and 95% confidence interval). Linear regression analyses of global cognition and AD biomarkers (independent variables) with dietary intake or REE (dependent variables)

*EN%* energy percentage, *REE* resting energy expenditure, *MMSE* Mini-Mental State Examination, *AD* Alzheimer’s disease, *Aβ*_*42*_ β-amyloid 42, *p-tau* phosphorylated tau

### Energy expenditure

REE was higher in patients with AD dementia and MCI (1704 ± 41 and 1754 ± 47 kcal/day) than in controls (1569 ± 34 kcal/day) (Table 2). There was no interaction with sex. REE per kg FFM was highest in patients with MCI and lowest in controls. Comparable results were found when restricting the analysis to participants with confirmed biomarker profile (Supplementary table B). Adjusted linear regression analyses showed no associations between MMSE score or AD biomarkers and REE (Table 3). Total time spent physically active did not differ across groups nor the time spent on physical activity at a certain intensity level (Table 2).

## Discussion

The main finding of this cross-sectional study is that patients with AD dementia and MCI had a higher REE than controls, despite a similar energy intake and physical activity level. These results suggest that the negative energy balance causing malnutrition in patients with AD dementia and MCI is more likely a consequence of a higher energy expenditure than of a reduced energy intake.

The higher REE of patients with AD dementia compared to controls confirms the results of one previous study [11]. We extend on this former study, by showing a higher REE in patients with MCI as well. In contrast to these findings, two other studies found similar REE in patients with AD dementia and controls [8, 12]. These two studies included patients with moderate and severe dementia (MMSE range 0–26), while we included patients with mild to moderate AD dementia (MMSE range 19–29), which might explain the discrepancies. Our results fit with the notion that REE is elevated in the beginning phase of the disease, as patients with MCI had the highest REE. Subsequently, REE declines in the more severe stages of the disease when FFM declines as well. Possibly, in the very early disease stages including MCI, cortical hypermetabolism as a compensatory mechanism causes higher energy needs of the brain [31] and therefore a higher REE. Another hypothesis is that the increased energy expenditure is caused by hypothalamic dysfunction, which has been showed in mice [32]. However, other underlying mechanisms, like elevated cortisol levels or stress, could not be excluded and need to be studied as well.

In contrast with former literature, diagnosis groups did not differ in physical activity [14, 15]. This is likely due to the wear location of the accelerometer, as previous studies used devices on the wrist (measuring mainly arm activity), while our device were attached to the waist and measured total body activity. Accelerometers worn at the hip or waist are generally more accurate than those worn on the wrist [33]. The average time the three groups spent physically active is comparable to Dutch older adults of the Longitudinal Aging Study Amsterdam (LASA), which also used accelerometers on the waist [34], indicating that physical activity data were, despite the small sample size, reliable.

In line with most previous studies, energy intake did not differ between patients with AD dementia and controls [8–10]. We additionally included a group of patients with MCI, who also showed a similar energy intake. Contrarily, one study observed a lower energy intake in patients with AD dementia than controls [7]. Discrepancies might be due to the use of two nonconsecutive 24-h recalls, which is a different method than we used and heavily relies on short term memory. We used two complementary methods to assess both habitual and actual dietary intake using an FFQ and food diary and observed a similar energy intake across methods. There were no associations of global cognition or AD biomarkers with energy intake, which further strengthens our finding that energy intake is not altered in mild to moderate AD. The mean energy intake of our population, around 2000 kcal, was higher compared to previous studies, ranging from 1500 to 1800 kcal, which is likely due to the higher age in these studies [7–10]. The energy intake of our population is comparable to the Dutch general older population [35, 36], indicating their dietary intake is reliable.

We investigated two components of energy balance (energy intake and energy expenditure), but other factors, like malabsorption, might also be important to explain malnutrition in AD patients and should be further investigated. For example, patients with AD dementia probably have a different microbiome than controls [37, 38]. Perhaps the altered microbiome affects nutrient uptake in the gut eventually leading to a negative energy balance. As unintended weight loss and malnutrition are associated with worse disease severity, understanding the malnutrition in dementia is important [39].

The strengths of this study include the use of two complementary dietary assessment methods and objective measurement of REE using indirect calorimetry in three, well diagnosed patient groups, including a group of patients with MCI. Dietary intake assessed by FFQ was available of a large sample compared to previous studies. We did not have CSF biomarkers of all participants and therefore performed a sensitivity analysis excluding participants without a matching biomarker profile. Results for dietary intake and REE did not change, indicating the results are reliable although diagnosis may not always be confirmed by CSF biomarkers. This study also has some limitations. First, because of the cross-sectional design, no causal inferences can be made. Future research should evaluate changes in body weight, energy intake, REE, and physical activity using a longitudinal design in order to investigate these aspects of energy balance across the different stages of the disease. Furthermore, we did not have any information about weight history, whether they lost or gained weight in the previous 6 months, which might hamper interpretation of an elevated REE. We currently follow our participants annually with neuropsychological assessments and body weight. Second, we do not have data of all our assessment methods for the total study sample. Participants with a valid measurement of REE (*n* = 88) were a selection of the FFQ sample (*n* = 219). Yet, participants with valid REE measurements did not differ in characteristics (sex, age, BMI and nutritional status) from the total sample. In addition, due to the small group with objective physical activity data available, analyses of REE could not be adjusted for physical activity, but were adjusted for fat-free mass instead. However, our data indicated no differences in physical activity between the diagnosis groups. For measurement of the REE, we used a standard-operating procedure, based on instructions by the manufacturer. The REE was continuously measured and the variability within the measurement was stable in all participants and did not differ across diagnosis groups. All participants were in rest during the measurement and no movements were observed. Lastly, in contrast to our expectations, diagnosis groups did not differ in BMI or MNA score. This is likely due to the small sample size. Given the higher REE and similar energy intake in patients with MCI and AD dementia, lower BMI and loss of body weight is expected. Unfortunately, we do not know the course of BMI or body weight of the participants before participating. Longitudinal studies are needed to investigate the REE and BMI change in relation to cognitive decline.

## Conclusions

Patients with AD dementia and MCI had a higher REE than controls, while they did not consume more energy. These results provide support for the notion that higher REE, rather than reduced energy intake, underlies the frequently observed malnutrition in patients with AD dementia. This could suggest that patients with AD dementia and MCI should adjust their dietary intake to compensate their higher energy needs.

## Supplementary information

**Additional file 1; Supplementary Table A.** Characteristics of study samples of 3-day food diary, REE and physical activity.

**Additional file 2: Supplementary Table B.** Dietary intake and energy expenditure according to diagnosis group, restricted to participants with a confirmed biomarker profile.

## Data Availability

The datasets used and/or analyzed during the current study are available from the corresponding author on reasonable request.

## Abbreviations

AD: Alzheimer’s disease
BMI: Body mass index
CSF: Cerebrospinal fluid
EN%: Energy percentage
FFM: Fat-free mass
FFQ: Food frequency questionnaire
MCI: Mild cognitive impairment
MMSE: Mini-Mental State Examination
MNA: Mini Nutritional Assessment
REE: Resting energy expenditure
SCD: Subjective cognitive decline
