# Supplementary material for: Energy intake and expenditure in patients with Alzheimer’s disease and mild cognitive impairment: the NUDAD project
Source: Alzheimers Res Ther. 2020 Sep 26;12:116. doi: 10.1186/s13195-020-00687-2 (PMC7520025; doi:10.1186/s13195-020-00687-2)
Supplement: Supplementary file 1 — Additional file 1; Supplementary Table A. Characteristics of study samples of 3-day food diary, REE and physical activity. [file 13195_2020_687_MOESM1_ESM.docx]

**Supplementary table A.** Characteristics of study samples of 3-day food diary, REE and physical activity

|  | **Controls** | **MCI** | **AD dementia** |
| --- | --- | --- | --- |
| *3-day food diary, N* | 40 | 22 | 30 |
| Age (y) | 62.5 ± 6.8 | 69.8 ± 7.2^a^ | 69.5 ± 9.4^a^ |
| Sex, female | 22 (55.0) | 6 (27.3) | 16 (53.3) |
| BMI (kg/m^2^) | 25.6 ± 4.0 | 25.1 ± 3.3 | 26.3 ± 4.9 |
| MNA score | 25.3± 1.2 | 24.7 ± 3.1 | 24.4 ± 2.5 |
| *Resting energy expenditure, N* | 38 | 22 | 28 |
| Age (y) | 62.7 ± 6.8 | 69.8 ± 7.2^a^ | 69.9 ± 9.5^a^ |
| Sex, female | 22 (57.9) | 6 (27.3) | 16 (55.2) |
| BMI (kg/m^2^) | 25.6 ± 4.1 | 25.1 ± 3.3 | 26.1 ± 4.9 |
| MNA score | 25.3 ± 1.2 | 24.7 ± 3.1 | 24.4 ± 2.5 |
| *Physical activity, N* | 11 | 10 | 10 |
| Age (y) | 64.0 ± 6.7 | 70.7 ± 8.6 | 67.3 ± 9.5 |
| Sex, female | 6 (54.5) | 2 (20.0) | 5 (50.0) |
| BMI (kg/m^2^) | 26.7 ± 5.5 | 25.2 ± 3.8 | 24.2 ± 2.4 |
| MNA score | 24.9 ± 1.1 | 24.2 ± 4.2 | 24.4 ± 1.7 |
| Number of valid days | 6.7 ± 0.6 | 6.6 ± 0.7 | 6.9 ± 0.3 |

Data in mean ± SD; n (%); AD= Alzheimer’s disease; MCI= mild cognitive impairment; BMI= body mass index; MNA= mini nutritional assessment without item on neuropsychological problems; ^a^ significantly different from controls upon post-hoc testing
