# Supplementary material for: Energy intake and expenditure in patients with Alzheimer’s disease and mild cognitive impairment: the NUDAD project
Source: Alzheimers Res Ther. 2020 Sep 26;12:116. doi: 10.1186/s13195-020-00687-2 (PMC7520025; doi:10.1186/s13195-020-00687-2)
Supplement: Supplementary file 2 — Additional file 2: Supplementary Table B. Dietary intake and energy expenditure according to diagnosis group, restricted to participants with a confirmed biomarker profile. [file 13195_2020_687_MOESM2_ESM.docx]

Supplementary table B. Dietary intake and energy expenditure according to diagnosis group, restricted to participants with a confirmed biomarker profile

|  | **Controls** | **MCI** | **AD dementia** | **P-value** |
| --- | --- | --- | --- | --- |
| **Dietary intake** |  |  |  |  |
| *FFQ, N* | 75 | 52 | 67 |  |
| Energy (kcal/d) | 2070 ± 72 | 2173 ± 82 | 2006 ± 74 | 0.312 |
| Protein (EN%) | 15.0 ± 0.3 | 15.5 ± 0.3 | 15.0 ± 0.3 | 0.456 |
| Carbohydrate (EN%) | 41.3 ± 0.8 | 41.2 ± 1.0 | 39.8 ± 0.9 | 0.443 |
| Fat (EN%) | 34.3 ± 0.7 | 34.6 ± 0.8 | 34.6 ± 0.7 | 0.952 |
| *3-day food diary, N* | 37 | 22 | 27 |  |
| Energy (kcal/d) | 2098 ± 73 | 2041 ± 92 | 1997 ± 85 | 0.691 |
| Protein (EN%) | 16.9 ± 0.5 | 15.8 ± 0.7 | 15.0 ± 0.6 | 0.064 |
| Carbohydrate (EN%) | 40.4 ± 1.3 | 42.6 ± 1.6 | 42.4 ± 1.5 | 0.504 |
| Fat (EN%) | 35.4 ± 1.1 | 35.8 ± 1.3 | 37.1 ± 1.2 | 0.596 |
| **Energy expenditure** |  |  |  |  |
| *Resting energy expenditure, N* | 36 | 19 | 24 |  |
| *Fasted (hours)* | 13.4 ± 0.7 | 14.6 ± 0.9 | 11.9 ± 0.8 | 0.072 |
| REE (kcal/d) | 1575 ± 36 | 1768 ± 48^a^ | 1702 ± 44 | **0.007** |
| REE (kcal/kg FFM) | 30.7 ± 0.7 | 34.5 ± 0.9 ^a^ | 33.4 ± 0.9 | **0.006** |

Data in mean ± SE; median [interquartile range]; intake: ANOVA adjusted for age, sex, education and BMI; REE: ANOVA post-hoc Bonferroni adjusted for age, sex, education and FFM. AD= Alzheimer’s disease; MCI= mild cognitive impairment; FFQ= food frequency questionnaire; EN% = energy percentage; FFM= fat free mass; REE= resting energy expenditure; ^a^ significantly different from controls upon post-hoc testing.
